# Association between asymptomatic hyperuricemia and new-onset chronic kidney disease in Japanese male workers: a long-term retrospective cohort study

**DOI:** 10.1186/1471-2369-12-31

**Published:** 2011-07-02

**Authors:** Masatoshi Kawashima, Koji Wada, Hiroshi Ohta, Hiroyuki Terawaki, Yoshiharu Aizawa

**Affiliations:** 1 Department of Occupational Health, Graduate School of Medical Sciences, Kitasato University School of Medicine, Sagamihara, Japan; 2 Department of Preventive Medicine and Public Health, Kitasato University School of Medicine, Sagamihara, Japan; 3 Division of Kidney and Hypertension, The Jikei University School of Medicine Kashiwa Hospital, Kashiwa, Japan

**Keywords:** chronic kidney disease, hyperuricemia, glomerular filtration rate, cohort, Japan

## Abstract

**Background:**

Hyperuricemia is prevalent in patients with chronic kidney disease (CKD). We explored the hypothesis that asymptmatic hyperuricemia may be associated with new-onset CKD.

**Methods:**

The participants were all male factory workers in Kanagawa, Japan (n = 1,285). All were over 40 years of age and had undergone annual health examinations from 1990 to 2007. Individuals with a history of gouty attacks were excluded from the study. A retrospective cohort study was conducted by following the estimated glomerular filtration rate (eGFR) for each participant over a maximum period of 18 years. The endpoint was new-onset CKD defined as eGFR < 60 mL/min/1.73 m^2^. The associations between new-onset CKD and the presence of hyperuricemia, low serum high-density lipoprotein cholesterol, hypertension, diabetes, and obesity were analyzed.

**Results:**

The mean (± standard deviation) follow-up period was 95.2 (± 66.7) months, and new-onset CKD was observed in 100 participants (7.8%) during this follow-up. Cox proportional hazards model revealed that the hazard ratio of new-onset CKD due to hyperuricemia, low serum high-density lipoprotein cholesterol, hypertension and obesity were 3.99 (95% confidence interval: 2.59-6.15), 1.69 (1.00-2.86), 2.00 (1.29-3.11) and 1.35 (0.87-2.10), respectively. Concerning hyperuricemia, low serum high-density lipoprotein cholesterol, hypertension and obesity, the log-rank tests showed *P *values of < 0.01, 0.01, < 0.01 and < 0.01, respectively.

**Conclusion:**

The results of this study suggest that asymptomatic hyperuricemia is a predictive factor for new-onset CKD for Japanese male workers.

## Background

End-stage renal disease (ESRD) poses a serious public health problem as a result of the costs of treatments such as dialysis and transplantation and harms the quality of life. Chronic kidney disease (CKD) precedes ESRD, and the progression of CKD to ESRD can be preventable by appropriate treatment. Prevention of new-onset CKD could effectively target ESRD. In 2002, the Kidney Disease Outcomes Quality Initiative of the National Kidney Foundation gave a definition and classification system for CKD, and CKD is defined as either glomerular filtration rate (GFR) < 60 mL/min/1.73 m^2 ^or kidney damage lasting for at least 3 months [1]. The estimated prevalences of CKD measured as GFR < 60 mL/min/1.73 m^2 ^are 8.1% in the United States [2] and 10.6% in Japan [3].

Previous studies have established hypertension [4-9] and diabetes [10-13] as predictive factors for CKD. Dyslipidemia [6,14,15], obesity [7,16] and hyperuricemia [17-20] have also been suggested to be predictive factors for CKD. However, it is not clear if asymptomatic hyperuricemia without gouty attacks is a risk factor for CKD. The aim of this study was to determine the associations between new-onset CKD and asymptomatic hyperuricemia, low serum high-density lipoprotein cholesterol (HDL-C), hypertension, diabetes and obesity in male factory workers over 40 years of age in Kanagawa, Japan.

## Methods

### Study Population

The participants were all male factory workers over 40 years of age who had undergone annual medical examinations from 1990 to 2007. This retrospective cohort study covered a maximum period of 18 years. To investigate the effects of asymptomatic hyperuricemia, 41 participants with a history of gouty attacks were excluded from the analysis. Participants with only one year of data, those lacking follow-up data, or those with incomplete data were also excluded.

### Disease Criteria

The presence of hyperuricemia, low serum HDL-C, hypertension, diabetes, and obesity were based on data from the participants' first-year medical examinations. Hyperuricemia was defined as a uric acid level of > 7.0 mg/dL. Low serum HDL-C was defined as HDL-C < 40 mg/dL. Hypertension was defined as a systolic blood pressure of ≥ 140 mmHg or diastolic blood pressure of ≥ 90 mmHg. Diabetes was defined as a fasting blood sugar level of ≥ 126 mg/dL. Obesity was defined as a body mass index (BMI) of ≥ 25 kg/m^2^.

### Outcomes

The endpoint was new-onset CKD. New-onset CKD was defined as the time point when estimated glomerular filtration rate (eGFR) fell below 60 mL/min/1.73 m^2^. The eGFR was calculated based on the participant's age and serum creatinine (Cr) level, using the equation:

determined by the Japanese Society of Nephrology [21]. In this study, serum Cr was measured using the Jaffe method from 1990 to 2002, and using the enzyme method from 2003 to 2007. Because the equation for estimating GFR used in this study is based on an enzyme method, eGFR for the period from 1990 to 2002 was calculated using the serum Cr level after subtraction of 0.2 mg/dL from the original serum Cr level [22]. Participants were excluded from the analysis if their earliest year's eGFR was < 60 mL/min/1.73 m^2^, if they had only 1 year's worth of data or if they lacked follow-up data.

### Statistical Analysis

The associations between new-onset CKD and the presence of hyperuricemia, low serum HDL-C, hypertension, diabetes, and obesity were analyzed. The covariates included age, hyperuricemia, low serum HDL-C, hypertension, diabetes and obesity. The follow-up period (in months) was the survival variable and new-onset CKD was a state variable. To adjust for age and the presence of disease as confounders, multivariate analysis was performed using Cox regression analysis [23]. A hazard ratio and 95% confidential interval (CI) were derived for each covariate. The hazard ratio was determined to be significant when the *P *value was < 0.05. In addition, Kaplan-Meier curves and log-rank tests were used to estimate the cumulative incidence of the covariates showing a significant hazard ratio [24]. The Japanese version of SPSS 17.0 for Windows was used for the analyses [25].

### Ethical Approval

This study was conducted with the approval of the ethics committee of Kitasato University School of Medicine.

## Results

The study involved 1,285 participants with a mean (± standard deviation [SD]) follow-up period of 95.2 (± 66.7) months. Of these participants, 100 (7.8%) developed CKD during the follow-up period, and their mean (± SD) follow-up period was 89.3 (± 62.0) months. The mean (± SD) follow-up period for participants who did not develop CKD was 95.7 (± 67.1) months. Table 1 shows the baseline characteristics of the participants in terms of age, levels of uric acid, HDL-C, systolic blood pressure, diastolic blood pressure, fasting blood sugar, and BMI. Hyperuricemia was detected in 166 participants (12.9%), low serum HDL-C in 153 (11.9%), hypertension in 255 (19.8%), diabetes in 51 (4.0%), and obesity in 255 (19.8%).

**Table 1:** Baseline characteristics of participants

	Participants (n = 1,285)	%
Age (yrs)		
40	433	33.7
41 - 45	273	21.2
46 - 50	276	21.5
51 - 55	220	17.1
≥ 56	83	6.5
Uric acid (mg/dL)		
> 7.0	166	12.9
≤ 7.0	1,119	87.1
HDL-C (mg/dL)		
< 40	153	11.9
≥ 40	1,132	88.1
Blood pressure (mmHg)		
SBP ≥ 140 or DBP ≥ 90	255	19.8
SBP < 140 and DBP < 90	1,030	80.2
Fasting blood sugar (mg/dL)		
≥ 126	51	4.0
< 126	1,234	96.0
Body mass index (kg/m^2^)		
≥ 25.0	255	19.8
< 25.0	1,030	80.2

HDL-C, high-density lipoprotein cholesterol

SBP, systolic blood pressure; DBP, diastolic blood pressure.

Table 2 shows the prediction for new-onset CKD during followed-up period. Of the participants with hyperuricemia at baseline, 32 (19.3%) developed CKD during the follow-up period. The results of Cox regression analysis revealed that the hazard ratio for new-onset CKD in the participants with hyperuricemia was 3.99 (95% CI: 2.59-6.15), showing a significant association between hyperuricemia and new-onset CKD. Likewise, the hazard ratio for new-onset CKD was 1.69 (95% CI: 1.00-2.86) in the participants with low serum HDL-C and 2.00 (95% CI: 1.29-3.11) in those with hypertension, indicating significant associations between new-onset CKD and these variables. However, a hazard ratio of 0.56 (95% CI: 0.17-1.77) indicated no significant association between new-onset CKD and diabetes. The hazard ratio was 1.35 (95% CI: 0.87-2.10) in the participants with obesity, indicating a weak association.

**Table 2:** Associations between predictors and new-onset CKD during a maximum period of 18 years follow-up

Predictors	Duration ± SD (months)	Cases	Incidence (%)	Hazard ratio	95% CI
Uric acid (mg/dL)					
> 7.0	76.3 ± 63.3	32	19.3	3.99	2.59, 6.15
≤ 7.0	98.0 ± 66.8	68	6.1	1.00	
HDL-C (mg/dL)					
< 40	89.3 ± 61.5	18	11.8	1.69	1.00, 2.86
≥ 40	96.0 ± 67.4	82	7.2	1.00	
Blood pressure (mmHg)					
SBP ≥ 140 or DBP ≥ 90	83.5 ± 62.8	33	12.9	2.00	1.29, 3.11
SBP < 140 and DBP < 90	98.1 ± 67.4	67	6.5	1.00	
Fasting blood sugar (mg/dL)					
≥ 126	95.1 ± 66.0	3	5.9	0.56	0.17, 1.77
< 126	95.2 ± 66.8	97	7.9	1.00	
Body mass index (kg/m^2^)					
≥ 25.0	93.2 ± 66.4	34	12.5	1.35	0.87, 2.10
< 25.0	95.7 ± 66.8	66	6.5	1.00	

CKD, chronic kidney disease; SD, standard deviation; CI, confidence interval;

HDL-C, high-density lipoprotein cholesterol; SBP, systolic blood pressure; DBP, diastolic blood pressure.

**Figure 1:**
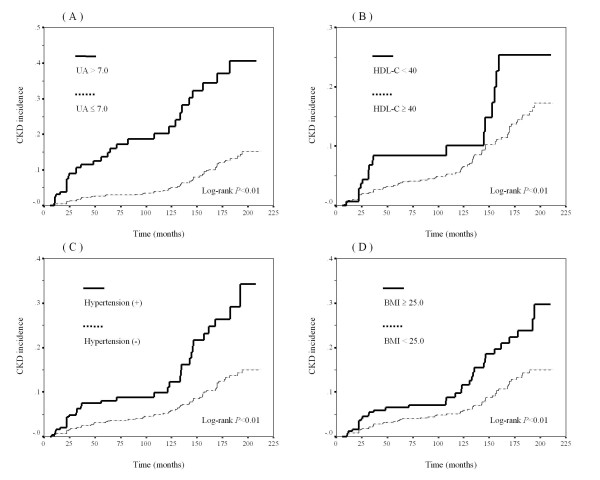
**Kaplan-Meier curves and Log-rank tests of CKD incidence**. The Kaplan-Meier curves show the follow-up periods (in months) and cumulative incidence rate of CKD. Solid lines represent cumulative incidence rates of CKD in participants with hyperuricemia (A), low serum HDL-C (B), hypertension (C) and obesity (D) while dotted lines represent for the CKD rate in participants without these factors. Log-rank tests were performed to determine differences in cumulative incidence rates. P values are shown in the figures. (A) UA > 7.0 mg/dL versus UA ≤ 7.0 mg/dL. (B) HDL-C < 40 mg/dL versus HDL-C ≥ 40 mg/dL. (C) (SBP ≥ 140 mmHg or DBP ≥ 90 mmHg) versus (SBP < 140 mmHg and DBP < 90 mmHg). (D) BMI ≥ 25.0 kg/m^2 ^versus BMI < 25.0 kg/m^2^. CKD, chronic kidney disease; UA, uric acid; HDL-C, high-density lipoprotein cholesterol; SBP, systolic blood pressure; DBP, diastolic blood pressure; BMI, body mass index.

The cumulative incidence of CKD was analyzed by the Kaplan-Meier method using the three variables (hyperuricemia, low serum HDL-C, and hypertension) showing significant associations and the one variable (obesity) showing a weak association with new-onset CKD in the Cox proportional hazards model (Figure 1). Concerning hyperuricemia, low serum HDL-C, hypertension and obesity, the log-rank tests showed *P *values of < 0.01 (Figure 1-A), 0.01 (Figure 1-B), < 0.01 (Figure 1-C) and < 0.01 (Figure 1-D), respectively.

## Discussion

We investigated the associations between new-onset CKD and asymptomatic hyperuricemia without gouty attacks, low serum HDL-C, hypertension, diabetes and obesity in Japanese male factory workers over 40 years of age. The results showed a significantly higher incidence of new-onset CKD in participants with asymptomatic hyperuricemia. A significantly higher incidence of new-onset CKD was also found in participants with low serum HDL-C and hypertension. The incidence of new-onset CKD tended to be higher, but not significantly, in obese participants, whereas no significant increase in the incidence of new-onset CKD was found in those with diabetes.

Several studies have reported possible associations between gouty attacks and renal function [26,27] and have suggested an association between hyperuricemia and CKD [17-20]. However, previous studies were not noticed about asymptomatic hyperuricemia without gouty attacks. Hyperuricemia in this study was defined as a uric acid level above 7.0 mg/dL without gouty attacks, and the hazard ratio for new-onset CKD in participants with hyperuricemia was 3.99, indicating a significant association. In addition, significant negative correlations were found between the uric acid values at the start of follow-up and the GFR values at the start of follow-up, after follow-up for 5 years, and after follow-up for 10 years (correlation coefficient: at the start of follow-up, -0.21 p < 0.001; after 5 years, -0.20 p < 0.001; and after 10 years:-0.22 p < 0.001) (data not shown). A uric acid level of 7.0 mg/dL is the diagnostic standard for hyperuricemia in Japan [28]. Obermayr et al stated in their report on 21,475 participants followed for 7 years, that the risk of new-onset CKD was increased by a factor of 1.74 in those whose uric acid levels were 7.0-8.9 mg/dL, and by a factor of 3.12 in those whose uric acid levels were ≥ 9.0 mg/dL [17]. Iseki et al. conducted a study in Japan and reported that the hazard ratio for progression to ESRD was 5.77 in women with uric acid levels of ≥ 6.0 mg/dL, showing a significant association, whereas there was no significant association between progression to ESRD and uric acid levels in men with uric acid levels of ≥ 7.0 mg/dL [19]. A follow-up study of patients with immunoglobulin A nephropathy for at least 8 years reported that serum creatinine (Cr) levels were significantly elevated in patients with hyperuricemia [20]. Hyperuricemia appeared to be affected by other lifestyle-related diseases such as hypertension, diabetes and dyslipidemia, but a significant association between hyperuricemia and new-onset CKD remained even after adjusting for factors related to hypertension, low serum HDL-C, obesity and diabetes. In this study, the hazard ratio for asymptomatic hyperuricemia was greater than those for other factors, including hypertension. Asymptomatic hyperuricemia was therefore suggested not only to be a predictive factor for CKD, but also to be a stronger predictor than established factors such as hypertension and diabetes.

Dyslipidemia is thought to be a risk factor for new-onset CKD. Previous studies detected associations between CKD and decreased HDL-C [6,7,14,15,29], increased total cholesterol [29,30], increased low-density lipoprotein cholesterol [14,28,30], and increased triglycerides [6,15]. However, several other studies found no such significant associations, and further investigation may be needed to clarify the association between dyslipidemia and CKD. Low serum HDL-C in this study was classified as HDL-C levels < 40 mg/dL, which is the diagnostic standard for low HDL-C in the United States (NCEP ATP III)[31] and in Japan (Japan Atherosclerosis Society; Guidelines for Prevention of Atherosclerotic Cardiovascular Diseases)[32]. When serum HDL-C was low, the incidence of new-onset CKD increased significantly (hazard ratio: 1.69), supporting an association between dyslipidemia and CKD.

An association between hypertension and renal disease has been established in a number of previous studies [4-9]. Increases in blood pressure have also been reported to be associated with increases in the incidence of ESRD [8,33,34], and progression of CKD can be prevented by antihypertensive treatment [5,8,9]. In this study, hypertension was defined as a systolic blood pressure of ≥ 140 mmHg or diastolic blood pressure of ≥ 90 mmHg. This is the standard classification for stage I hypertension in the United States (JNC7)[35] and mild hypertension in Japan (JSH2009)[36] and is used as the diagnostic standard for hypertension. The hazard ratio for new-onset CKD in our study was 2.00, showing a significant association with hypertension. These results are consistent with those from several previous studies that also found an association between hypertension and CKD.

Diabetes is an established predictor of renal disease [10-13]. However, we found no significant association between diabetes and new-onset CKD. GFR decreases with the duration of diabetes, but it has been suggested that glomerular hyperfiltration occurs during the early stage of diabetes, leading to a temporary increase in GFR [37]. The mean (± SD) follow-up period was 95.1 (± 66.0) months in our participants with diabetes, but it is possible that the GFR was increased or not markedly decreased in some participants who were still in the hyperfiltration phase. Thus, it is necessary to determine the presence of new-onset CKD employing urinary albumin measurement in participants with diabetic nephropathy during the early stage. Diabetes reportedly did not significantly affect the progression of CKD in Japanese participants; significant reductions in GFR might not have been observed because of hyperfiltration, as observed in this study [6].

Obesity has been suggested to be a risk factor for ESRD [7,16]. Kramer et al. reported that the risk of new-onset CKD was increased by a factor of 1.21 in participants whose BMI was 25.0-29.9 kg/m^2 ^and by a factor of 1.40 in those whose BMI was ≥ 30 kg/m^2^[38]. However, a study conducted in Japanese participants found that a BMI of ≥ 25 kg/m^2 ^in male participants had no effect on the progression of CKD to stage III or more severe disease [6]. In our study, a BMI of 25 kg/m^2 ^was used as the standard for defining obesity, according to the criteria for obesity in Japan (Guidelines for the Treatment of Obesity, 2006)[39]. The hazard ratio for new-onset CKD in participants with obesity was 1.35, showing no significant association; however, a weak association was observed, since the 95% CI was 0.87-2.10. Further studies with longer follow-up periods in more participants are needed to clarify the association between obesity and new-onset CKD.

In this study, new-onset CKD was defined as GFR < 60 mL/min/1.73 m^2^. It is therefore important to clarify the methods used for estimating the GFR; GFR-estimating equations for Japanese devised by the Japanese Society of Nephrology in March, 2008 [21]. Imai et al. previously reported a different equation for estimating GFR in Japanese participants [40], but it has been suggested that this might underestimate GFR values. The equation used in the current study was a revised version of the previous equation, and might produce more accurate estimates of GFR in the Japanese population. According to the diagnostic criteria for CKD, abnormal urinalysis results, such as proteinuria, and abnormal findings on echography, can also be used to diagnose CKD. However, in line with many previous studies, the current study defined CKD as GFR < 60 mL/min/1.73 m^2^.

Hyperuricemia, low serum HDL-C, hypertension, diabetes, and obesity were defined in this study based on the first year's data for each participant. Treatment for any of these diseases was not taken into account, except for the exclusion of any individuals with a history of gouty attacks. It is therefore possible that some of the participants who were classified as a uric acid level of ≤ 7.0 mg/dL were actually receiving treatment. In this case, compared with strictly extracting participants who have treatment of hyperuricemia, the power of statistically significance of the association between hyperuricemia and CKD could be low. However, hyperuricemia could be a predictive factor for new-onset CKD in this study, as suggested by the significant association between hyperuricemia and new-onset CKD. Conclusions

The results of this study suggest that asymptomatic hyperuricemia without gouty attacks is a predictive factor for new-onset CKD. Therefore, the appropriate treatment might reduce the number of patients of CKD.

## Abbreviations

ESRD: end-stage renal disease; CKD: chronic kidney disease; GFR: glomerular filtration rate; HDL-C: high-density lipoprotein cholesterol; BMI: body mass index; eGFR: estimated glomerular filtration rate; Cr: creatinine; CI: confidence interval; SD: standard deviation.

## Competing interests

The authors declare that they have no competing interests.

## Authors' contributions

MK and KW designed the study, and completed the manuscript. HO, HT and YA undertook statistical analyses and assisted with drafting the manuscript from a clinical perspective. All authors read and approved the final manuscript.

## Pre-publication history

The pre-publication history for this paper can be accessed here:

http://www.biomedcentral.com/1471-2369/12/31/prepub
